# Candida Interactions with the Oral Bacterial Microbiota

**DOI:** 10.3390/jof4040122

**Published:** 2018-11-03

**Authors:** Daniel Montelongo-Jauregui, Jose L. Lopez-Ribot

**Affiliations:** Department of Biology, South Texas Center for Emerging Infections Diseases, The University of Texas at San Antonio, San Antonio, TX 78249, USA; daniel.montelongo@utsa.edu

**Keywords:** oral microbiota, *Candida albicans*, biofilms, interkingdom interactions

## Abstract

The human oral cavity is normally colonized by a wide range of microorganisms, including bacteria, fungi, Archaea, viruses, and protozoa. Within the different oral microenvironments these organisms are often found as part of highly organized microbial communities termed biofilms, which display consortial behavior. Formation and maintenance of these biofilms are highly dependent on the direct interactions between the different members of the microbiota, as well as on the released factors that influence the surrounding microbial populations. These complex biofilm dynamics influence oral health and disease. In the latest years there has been an increased recognition of the important role that interkingdom interactions, in particular those between fungi and bacteria, play within the oral cavity. *Candida* spp., and in particular *C. albicans*, are among the most important fungi colonizing the oral cavity of humans and have been found to participate in these complex microbial oral biofilms. *C. albicans* has been reported to interact with individual members of the oral bacterial microbiota, leading to either synergistic or antagonistic relationships. In this review we describe some of the better characterized interactions between *Candida* spp. and oral bacteria.

## 1. Introduction

Contemporary microbiome studies indicate that that over 700 species of microorganisms colonize the human oral cavity (http://www.homd.org/). Given what an astoundingly microbe-rich niche the oral cavity is, the study of such a complex collection of microorganisms is very challenging. Among the great diversity of microorganisms found as residential microbiota, both in health and disease, there are representatives of the three domains of life: Archaea [1,2,3], Bacteria [1,2], and Eukarya [3,4]. Presence of microorganisms varies among individuals, reflecting differences in diet, sampling times of day, health status, and geographical locations, and also varies according to the microenvironment within the oral cavity under consideration (i.e., teeth, saliva, tongue, gingiva, and other epithelial surfaces of the oral mucosae) [5,6]. It is widely accepted that most microorganisms are found within multispecies biofilms, complex microbial communities with cooperative behavior covering the different surfaces within the oral cavity. Fortunately, with the emergence of newer technologies such as high throughput sequencing, a more thorough analysis can be performed to identify the many microorganisms present at a particular site. In this regard, efforts have been made to characterize what would be considered a healthy oral microbiota compared to that commonly associated with disease [7,8,9]. In healthy individuals, the pH of saliva is kept relatively neutral, which permits the growth of many microorganisms at a physiological temperature [10]. Early colonizers, predominantly streptococci and *Actinomyces* spp., bind to oral tissues via nonspecific interactions with charged surfaces in the host that are bathed with saliva, as well as with proteins and glycoproteins such as mucin in the same saliva, which, along with other metabolites (pellicle formation), promote adherence of the early colonizers, which in turn allows co-adherence of secondary colonizers [11,12,13]. Furthermore, saliva contains secretory IgA (s-IgA) which can inhibit bacterial attachment to dental surfaces and oral tissues [14]; for example caries-free patients tend to have higher concentration of s-IgA [15]. To overcome s-IgA and adhere to dental tissues, the early colonizer *Streptococcus sanguinis* is capable of producing IgA proteases [16], depleting s-IgA and permitting its adherence to host tissues in the oral cavity [17]. Also, early colonizers express a number of adhesins in their cell surface that are important for specific and nonspecific adherence to dental surfaces and other microorganisms. In this manner, antigen I/II (AgI/II) family polypeptides in oral streptococci [18,19] have been described to mediate adhesion to oral tissues, as well as to other bacteria and fungi including *A. naueslundii* [20], *P. gingivalis* [21], and *C. albicans* [22].

Of increasing interest are interkingdom interactions within the oral microbiota, such as those established between fungi and bacteria [3,23,24]. Particularly intriguing are those interactions that lead to an increased pathogenicity of either microorganism involved [25,26]. The opportunistic pathogen *Candida albicans* is the most frequent fungus isolated during oral infections, and a number of studies have shown that different oral bacteria adhere to *C. albicans* in oral biofilms and can modulate its pathogenicity [27,28]. Moreover, these interactions are described to be multidirectional because the presence of *C. albicans* or other *Candida* spp. can also influence the behavior of the bacterial microbiota [29]. Therefore, one has to consider the sum of interactions between all the microorganisms involved, which ultimately gives rise to a tremendous degree of complexity associated with these multispecies oral biofilms. In this review we elaborate on some of the best characterized interactions between individual oral bacteria and *Candida* spp., with special emphasis on *C. albicans*, the fungal species most commonly isolated from the oral cavity.

## 2. *C. albicans* Interactions with Oral *Streptococci*

### 2.1. Mutans Group Streptococci

#### *Streptococcus* *mutans*

*Streptococcus mutans*, the etiological agent of early childhood caries (ECC) [30], has been described to intimately communicate with *C. albicans* in a complex bidirectional interaction [25]. It has been thoroughly described that carbohydrate consumption, in particular sucrose is closely linked to the development of tooth decay [31]. In fact, long-term excessive carbohydrate uptake can lead to a cariogenic microbiota, lowering pH, and increasing the presence of mutans streptococci such as *S. mutans*, while the presence of noncariogenic streptococci under these conditions is diminished [32,33]. Aside from sucrose, *S. mutans* feeds off various other carbohydrate sources such a glucose, fructose, mannose, and other fermentable sugars via the phosphotransferase system (PTS) that lead to production of exopolymeric substance (EPS) in the biofilm matrix and acid, which is common in the formation of dental caries [34,35]. A well-recognized mechanism for the breakdown of sucrose is through the secretion of the exoenzymes known as glucosyltranferases (Gtfs), which catalyze the transfer of glucosyl units formed following the cleavage of sucrose to a growing α-glucose chain as glucan polymers assemble, also producing fructose in the process [36]. It has been demonstrated in vitro that high levels of sucrose can lead to increased interactions between *S. mutans* and *C. albicans*, resulting in dramatically enhanced microbial burden and production of biofilm matrix, which could potentially have important clinical repercussions in the production of severe childhood caries [37,38,39]. In particular as it relates to interactions with *C. albicans*, GtfB helps the fungus by producing carbohydrate that can then be metabolized by *C. albicans* [40]. Interestingly, GtfB can also bind directly to the surface of *C. albicans*, allowing it to adhere to dental surfaces and leading to the formation of mixed biofilms [41]; additionally, *S. mutans* GtfB has been shown to upregulate the expression of *HWP1*, *ALS1*, and *ALS3* genes encoding important adhesins in *C. albicans* [42]. Furthermore, an RNA-Seq study from co-cultures showed that the presence of *C. albicans* enhances sugar metabolism pathways of *S mutans* [43]. Using *C. albicans* mutants in vitro, Hwang et al. demonstrated that mannans on the fungal cell wall have an important role in the direct binding with GtfB, as they observed reduced interaction with *C. albicans* mutants deficient in *O*-mannan and *N*-mannan outer chain, *Δpmt4* and *Δoch1*, respectively, which resulted in poor mixed biofilm formation [44]. Confirmation of these observations were performed in an in vivo rodent model of dental caries, showing that the *C. albicans Δoch1* mutant was deficient in adhesion to tooth surfaces of rats even in the presence of *S. mutans* [44]. Furthermore, adherence of GtfB and production of EPS has been reported to increase *C. albicans* resistance to fluconazole treatment, and disruption of EPS by povidone iodine in combination with fluconazole rendered the antifungal treatment more effective [45]. Moreover, the addition of the bacterium *Lactobacillus salivarius* in multispecies biofilms can inhibit in vitro biofilm formation of both *S. mutans* and *C. albicans* [46].

Confounding matters for the classification of this relationship as always synergistic are secreted factors detected on spent media of *S. mutans* cultures that negatively affect growth and pathogenetic characteristics of *C. albicans*. Quorum sensing molecules, such as the competence-stimulating peptide (CSP) [47], trans-2-decenoic acid [48], and mutanobactin A [49], have been reported to inhibit filamentation and induce *C. albicans* yeast formation. Interestingly, presence of *C. albicans* can influence the expression of those factors in *S. mutans* as Sztajer et al. were able to detect stimulation of quorum-sensing using spent media from dual-species biofilms [50]. The authors reported the spent media from a co-culture to induce expression of quorum sensing regulon, inducing CSP and XIP (alternative sigma factor *sigX*-inducing peptide) important for induction competence in *S. mutans*, as well as production of mutacins and fratricins [50]. Additionally, the fungal quorum sensor farnesol was also shown to enhance *S. mutans* biofilm formation, microcolony development, and GtfB activity at low concentrations [51]; while proven to have antibacterial properties at high concentrations [52].

Furthermore, in a recent publication, a member of the AgI/II family polypeptides, which was first identified in *S. mutans* [53], SpaP, has been found to be important direct interaction with *C. albicans* in vitro, and to facilitate colonization of both microorganisms in a *D. melanogaster* in vivo model [54].

### 2.2. Mitis Group Streptococci

Many members of the mitis group of streptococci have been identified to interact with *C. albicans* [55,56]. This group consists mostly of early colonizers of the oral microbiota, and given their promiscuity at binding different surfaces and microorganisms, they are considered initial settlers of dental plaque [57]. The mitis group comprises 13 species of streptococci including four species, namely *S. gordonii*, *S. mitis*, *S. oralis*, and *S. sanguinis*, for which existing evidence indicates their ability to interact directly with *C. albicans* [55]. Here we will list some of the most relevant interactions between *C. albicans* and the mitis group members.

#### 2.2.1. *Streptococcus* *gordonii*

**Figure 1:**
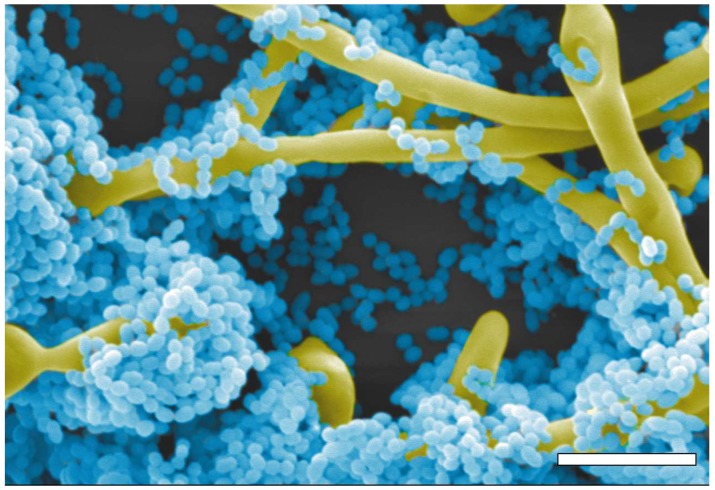
Pseudo-colored image of a scanning electron micrograph of mixed biofilms of *C. albicans* SC5314 (yellow) and *S. gordonii* DL1.1 (blue). Scale bar is 5 μm.

Existing evidence points out to the various ways in which these two microorganisms, *Streptococcus gordonii* and *C. albicans*, interact with one another (Figure 1), with most information coming from the streptococcal side. These interactions have reported to be mostly synergistic [28,58,59,60], and have revealed to be through both direct physical contact and the release of diffusible factors [58]. Various in vitro models have been designed to study their interactions including biofilms grown on microtiter plates with or without presence of an artificial salivary pellicle [58,59], titanium implant material [60], and biofilms grown on mucosal tissue analogs [28]. Streptococcal adhesins CshA as well as SspA and SspB (belonging to the AgI/II family polypeptides) have been identified to be important for the direct binding to fungal cells; in particular SspB was shown to directly bind to hyphal-specific adhesins Als3, Eap1, and Hwp1 [22,58,61,62]. The same group tested the regions of Als3 responsible for the binding to *S. gordonii,* concluding that deletion of amino acids in the N-terminal region of the protein significantly reduced their interaction [61]. It was also demonstrated that *C. albicans* mutant strains defective in *O*-mannosylation, such as *Δmnt1* and *Δmnt2* mutants, do not show a strong direct association with *S. gordonii* [63], with follow-up studies demonstrating a role for secreted aspartyl protease 9 (Sap9) during the interaction of both microorganisms by modulating cell surface hydrophobicity and the expression of other surface adhesins, most notably Eap1 [64].

With respect to the communication of these microorganisms via released factors, Bamford et al. tested this interaction using an *S. gordonii ΔluxS* mutant, which is a gene important for the production of autoinducer 2 (AI-2), a quorum sensing factor released and recognized by a great number of bacterial species that permit their cross-talk [58,65,66]. The authors observed reduced biofilm biomass in the presence of this mutant as well as less hyphal formation by *C. albicans.* Interestingly, they also observed that the presence of *S. gordonii* inhibited the effect of addition of exogenous farnesol to *C. albicans*, showing that the likely mechanism by which *S. gordonii* can induce *C. albicans* filamentation is by blocking the effect of farnesol repression at later stages of biofilm formation [58]. Ricker et al. observed that *S. gordonii* expression of glucosyltransferase G (GtfG) also contributes to coaggregation with *C. albicans* [67] in a very similar fashion to what was described in the case of *S. mutans* [41,44,68]. Finally, the growth of both microorganisms in the form of mixed biofilms also appears to be relevant in their increased resistance to antimicrobial treatment [59,60].

Similarly to what is observed with *S. mutans*, CSP produced by *S. gordonii* can modulate mixed biofilm formation, as it was demonstrated that the *S. gordonii* comCDE (competence) operon influences the formation of mixed biofilms with *C. albicans*, mostly by modulating the production of eDNA and the incorporation of fungal cells within mixed biofilms [69].

#### 2.2.2. *Streptococcus* *oralis*

Similar to *C. albicans*, *S. oralis* is mostly considered an oral commensal; however, it is also an opportunistic pathogen in immunocompromised individuals like cancer and cystic fibrosis patients [70,71,72,73]. Together, these two microorganisms were shown to form stronger biofilms when mixed compared to their respective single-species counterparts [28]. Diaz et al. observed strong biofilm formation and increased invasion in a mucosal tissue analog compared to the individual species, suggesting that they synergize during infection [28]. This synergism was corroborated using an in vivo murine model where both microorganisms were given orally to immunosuppressed mice. Results indicated higher streptococcal burden and colonization in the presence of *C. albicans*, demonstrating that *S. oralis* benefits from the presence of *C. albicans* in vivo [74]. The authors also observed an exacerbated inflammatory response during mixed infection that was dependent of TLR2 signaling [74]. The same group demonstrated that biofilm formation with these mixed microorganisms is higher in moist compared to dry environments, and that high nutrient availability increases tissue invasiveness [75]. *C. albicans* pseudohyphal mutants displayed decreased interaction with *S. oralis* compared to *C. albicans* cells that can produce true hyphae [75].

In recent publications, Xu et al. showed that, both in vitro and in vivo, *S. oralis* and *C. albicans* co-infection leads to an increased invasion of mucosal tissues due in part to the release of μ-calpain, which degrades E-cadherin junctions of epithelial cells, permitting access to both fungal and bacterial cells [76]. *S. oralis* is also capable of inducing *C. albicans* filamentation through the master regulator *EFG1* [70]. Finally, a new in vivo model was recently developed for the study of these interactions using a clinically relevant murine model that mimics chemotherapy-induced mucositis after treatment with 5-fluorouracil [71,72].

#### 2.2.3. *Streptococcus sanguinis, Streptococcus parasanguinis*, *and Streptococcus mitis*

There have been reports that *S. mitis*, *S. sanguinis*, and *S. parasinguinis* are able to interact with *C. albicans* [28,56,73,77,78,79]. *S. sanguinis* has been reported to synergize with *C. albicans* during biofilm formation [28]. Previously, *S. sanguinis* had been recognized to produce a bacteriocin that has antibacterial properties [80]. Ma et al. tested the effect a bacteriocin against oral pathogens such as *P. gingivalis* and *Prevotella intermedia* and fungal species *C. albicans* and *C. tropicalis* [77]. The authors observed that intracellular proteins from an extract produced by *S. sanguinis* in culture medium displays antibacterial activity against as *P. gingivalis* and *P. intermedia,* as well as inhibitory effects on growth and filamentation of both *Candida* spp. [77], thereby reflecting the important role that *S. sanguinis* plays in the oral microbiota. Despite of this, the sum of all stimuli in multispecies biofilms using *S. sanguinis* and other oral bacteria promoted virulence of *C. albicans* in acrylic surfaces [78] and titanium [79]. Novel treatments against these resilient mixed biofilms have been described, including some promising results using photodynamic inactivation [81].

### 2.3. Salivarius Group Streptococci

#### *Streptococcus* *salivarius*

The *S. salivarius* probiotic strain *K12* inhibits adherence and filamentation of *C. albicans*, and in a murine model of infection candidiasis protected mice from severe candidiasis [82]. The effects were not fungicidal and may be different than those displayed as a probiotic against other bacterial species, which are mostly mediated by the antimicrobial activity of bacteriocin-like inhibitory substances.

## 3. *C. albicans* Interactions with Other Oral Bacteria

### 3.1. Porphyromonas gingivalis

*C. albicans* has been reported to interact with the pathogenic periodontal bacterium *Porphyromonas gingivalis*, a major etiological agent of chronic periodontitis [83]. Interestingly, similar to what is observed in the mitis group of streptococci, hyphal-specific adhesin Als3 on the fungal surface is considered to be important for the interaction with *P. gingivalis* [84], and pretreatment of gingival epithelial cells and fibroblasts with *C. albicans* increased *P. gingivalis* invasion [85]. Interestingly, *S. gordonii* can bind directly to and communicate with *P. gingivalis* via secreted factors, exacerbating its pathogenicity leading to periodontal disease [86,87,88]. In addition, as previously discussed, *S. gordonii* can be considered an aide of *C. albicans* attachment and filamentation in oral tissues. Thus, this three-way Candida–Streptococcal–Porphyromonas interaction represents an intriguing example of the complexity associated with oral microbiota studies. Moreover, Haverman et al. used an in vitro assay that demonstrates that *P. gingivalis* can delay oral epithelium cell migration when interacting with other *Candida* spp. such as *C. kefyr* and *C. glabrata* [89].

### 3.2. Actinomyces spp.

Along with various species of streptococci, *Actinomyces* spp. are among the first colonizers of oral surfaces and tissues [90,91,92]. Various *Actinomyces* spp., including *A. viscosus* [73,78,93], *A. naeslundii* [93,94,95,96], *A. odontolyticus* [78], and *A. oris* [97,98], have been described to co-aggregate with *C. albicans.* Evidence has shown that this interaction is not necessarily contact-dependent, as some metabolites produced by *A. naeslundii* and *A. viscosus* inhibit *C. albicans* growth at high concentrations and boost its growth at lower concentrations [93]. A second report showed that *A. viscosus*, *A. naeslundii*, and *A. odontolyticus* cell suspensions, supernatants, and bacterial lysates were able to inhibit growth of *C. albicans* [99]. Additionally, *C. albicans* adhesion to buccal epithelial cells and hyphal formation was reduced, while production of phospholipase C was increased, after incubation with supernatants from *Actinomyces* spp. [99]. It has also been reported that *C. albicans* coaggregation with *A. viscosus*, *A. naeslundii*, and *A. odontolyticus* appears to depend on the fungal strain [95] and medium of growth [96]. There are previous reports that *Actinomyces* spp. can also co-aggregate with oral streptococci [91,92] in particular with *S. oralis* [91], which in turn suggests that *C. albicans* could form a triad with both early colonizers. Interestingly, publications from Cavalcanti et al. highlight the potential of *A. oris* to incorporate into mixed biofilms with *S. oralis* and *C. albicans* on denture material [97] and salivary pellicles [98]. In biofilms grown on acrylic resin, the authors observed synergism and collaboration in the triad mixed biofilms, despite the fact that in dual species biofilms the biovolume of *C. albicans* only discretely augmented in the presence of *A. oris* [97]. Also, they demonstrated that mutants of *A. oris* lacking type 1 and 2 fimbriae were not able to bind to *S. oralis* but were still able to adhere to *C. albicans* in the mixed biofilms [98].

### 3.3. Fusobacterium nucleatum

The common oral commensal *Fusobacterium* spp. have also proved to be opportunistic pathogens associated with a very diverse range of diseases, from oral and gastrointestinal infections to adverse pregnancy outcomes [100]. Interestingly, many *Fusobacterium* species have been demonstrated to adhere to *C. albicans* [101] and *C. dubliniensis* [102]. Work from Wu et al. revealed that coaggregation of *C. albicans* and *F. nucleatum* is partly the result of expression of *C. albicans FLO9* encoding an adhesin-like cell wall mannan and *F. nucleatum* radD encoding an arginine-inhibitable adhesin; although the authors could not conclude if these two factors interact directly with one another in their in vitro assay [103]. Moreover, the interaction can be inhibited by the addition of mannose or arginine to wild-type strains [103]. Interestingly, *F. nucleatum* expression of radD along with aid1 has also been described to be important in the coaggregation of oral streptococci and both adhesins can be inhibited by the addition of arginine [104,105]. In a follow-up publication, the same group concluded that both microorganisms attenuate virulence of one-another mutually in a contact-dependent manner: the macrophage-killing ability of *C. albicans* was reduced because *F. nucleatum* inhibits filamentation, while the yeast also repressed *F. nucleatum*-induced macrophage responses [106]. The authors concluded that this mutualistic attenuation of virulence may benefit both microorganisms by promoting long-term commensalism within the oral cavity.

### 3.4. Rothia dentocariosa

Considered an emerging opportunistic bacterium, *Rothia dentocariosa* has been reported to be commonly isolated together with *C. albicans* from failed silicone voice prostheses in patients that underwent a laryngectomy [107] and patients that required frequent replacement of such prostheses [26,108]. Millsap et al. tested used various bacteria in combination with *Candida* spp. on silicone rubber surface for their ability to enhance fungal colonization, concluding that *R. dentocariosa*, *S. aureus*, and *S. mitis* aided colonization of *C. albicans* and *C. tropicalis* [109]; the same species also worsened air flow resistance in these devices [110]. However, despite this increase in adhesion, the same group also reported that the bacterium reduced filamentation and overall biofilm formation by *C. albicans* [108]. Uppuluri et al. tested the interaction of *R. dentocariosa* with *C. albicans* in vitro using suspension cultures and growing mixed biofilms on silicone rubber [111]. The authors observed delayed planktonic growth and decreased filamentation in *C. albicans* biofilms [111]. Furthermore, biomass in mixed-biofilms was reported to be 2-fold lower compared to monospecies *C. albicans* biofilms [111]. These observations were corroborated by determining global transcriptional profiles of mixed biofilms, in which *C. albicans* genes involved in regulation of filamentation as well as hyphal-specific genes were downregulated as compared to expression levels detected in monospecies *C. albicans* biofilms [111]. Interestingly despite of the transcriptional data, the group observed that the hyphal-specific adhesin Als3 was important for the interaction of both microorganisms [111].

### 3.5. Aggregatibacter actinomycetemcomitans

An oral commensal and opportunistic pathogen [112], *Aggregatibacter actinomycetemcomitans* was recently included in the list of oral bacteria that can interact with *C. albicans* [113]. Despite being the only report on this interaction to date, Bacthiar et al. observed the effect by a common factor used by many oral bacteria, AutoInducer-2 (A1-2) [113]. Recently, AI-2 was described to be required for biofilm formation of *A. actinomycetemcomitans* [114]. *A. actinomycetemcomitans* is able to inhibit biofilm formation by *C. albicans* in co-cultures but this effect is rescued using a *ΔluxS* mutant strain that cannot produce AI-2 [113]. Additionally, spent medium from wild-type *A. actinomycetemcomitans* was also able to inhibit biofilm formation and disrupt *C. albicans* preformed biofilms [113]. Intriguingly, AI-2 from *A. actinomycetemcomitans* inhibits *C. albicans* hyphal formation, while the same factor produced by *S. gordonii* [58] has the opposite effect, suggesting differences between the factors released by each bacterial species.

### 3.6. Staphylococcus aureus

An opportunistic pathogen and one of leading causes of nosocomial infections, *S. aureus* is also commonly isolated from the oral cavity of healthy individuals [115,116] as well as from patients with oral infections that are also commonly associated with the presence of *C. albicans*, such as denture stomatitis [117,118] and angular cheilitis [119,120,121,122]. *S. aureus* is thought to benefit from the presence of *C. albicans.* Using in vitro models, Peters et al. described the *S. aureus* ability adhere to Als3 in hyphae [123]. Using a co-colonization oral murine model Schlecht et al. observed a synergistic infection by both *C. albicans* and *S. aureus*. However, if mice were inoculated with an *Δals3 C. albicans* mutant strain, despite the detection of colonization in tongues of the animals, *S. aureus* could not get into the bloodstream and cause disseminated infection [124]. Recently, a study showed increased production and an important role for eDNA in these mixed biofilms, with the authors concluding that eDNA supported *S. aureus* adherence in the dual-species biofilms [125]. It was also demonstrated that the presence of *C. albicans* or its secreted cell wall polysaccharide material in mixed biofilms significantly enhanced the tolerance of *S. aureus* to antibacterial antibiotics [126].

### 3.7. Enterococcus faecalis

There is growing evidence for the coexistence between enterococci and *C. albicans*, including within the oral cavity [127,128,129]. For example, *E. faecalis* and *C. albicans* were co-isolated in approximately 10% of root canal and periapical infections, and in approximately 40% of oral mucosal lesions [129]. Another retrospective study found *E. faecalis* among the bacterial microbiota most often accompanying *Candida* spp. in oral samples [127]. Of note, a recent report indicates that *E. faecalis* can inhibit *C. albicans* filamentation, biofilm formation, and overall virulence [130]. Further experiments demonstrated that a secreted bacterial compound was responsible for the inhibition of hyphal growth in dual-species biofilms, and that this inhibition was partially dependent on the Fsr quorum-sensing system, a major regulator of virulence in this opportunistic pathogenic bacterium [131].

## 4. Non-*albicans Candida* Species in the Oral Microbiome

There is an increased recognition of the role that non-albicans Candida species (NACS) play in the oral cavity both as commensals and etiological agents of infection, including some of the interactions among different Candida species [132,133,134,135,136,137,138,139]. Unfortunately, very little is known about NACS interactions with oral bacteria, as most focus has been placed on *Candida albicans*. For example *C. dubliniensis* has been described to interact with *Fusobacterium nucleatum* [102], and a clinical isolate of *C. tropicalis* was reported to be capable of coaggregating with different oral bacteria including *F. nucleatum*, *A. viscosus*, oral streptococci, and *L. amylovorous* [140]. Clearly, more work is needed in order to elucidate the NACS interactions with oral bacteria both in health and disease.
